# New risk prediction model of coronary heart disease in participants with and without diabetes: Assessments of the Framingham risk and Suita scores in 3-year longitudinal database in a Japanese population

**DOI:** 10.1038/s41598-019-39049-w

**Published:** 2019-02-26

**Authors:** Hiroyuki Hirai, Koichi Asahi, Satoshi Yamaguchi, Hirotaka Mori, Hiroaki Satoh, Kunitoshi Iseki, Toshiki Moriyama, Kunihiro Yamagata, Kazuhiko Tsuruya, Shouichi Fujimoto, Ichiei Narita, Tsuneo Konta, Masahide Kondo, Yugo Shibagaki, Masato Kasahara, Tsuyoshi Watanabe, Michio Shimabukuro

**Affiliations:** 1 Department of Diabetes, Endocrinology and Metabolism of Medicine, Fukushima Medical University, 960-1295 Fukushima City, Fukushima Japan; 2 Department of Internal Medicine, Shirakawa Kosei General Hospital, Shirakawa City, 961-0005 Fukushima Japan; 3 Steering Committee of Research on Design of the Comprehensive Health Care System for Chronic Kidney Disease (CKD) Based on the Individual Risk Assessment by Specific Health Check, 960-1295 Fukushima, Japan; 4 Department of Cardiology, Nakagami Hospital, 610 Noborikawa, 904-2142 Okinawa Japan; 5 Department of Metabolism and Endocrinology, Juntendo University School of Medicine, Bunkyo, 113-8421 Tokyo Japan; 6 Department of Internal Medicine, Fukushima Rosai Hospital, Iwaki City, 973-8403 Fukushima Japan

## Abstract

The Framingham Risk Score (FRS) has been reported to predict coronary heart disease (CHD), but its assessment has been unsuccessful in Asian population. We aimed to assess FRS and Suita score (a Japanese CHD prediction model) in a Japanese nation-wide annual health check program, participants aged 40–79 years were followed up longitudinally from 2008 to 2011. Of 35,379 participants analyzed, 1,234 had new-onset CHD. New-onset CHD was observed in diabetic men [6.00%], non-diabetic men [3.96%], diabetic women [5.51%], and non-diabetic women [2.86%], respectively. Area under the curve (AUC) of receiver operating characteristic (ROC) curve for CHD prediction were consistently low in Suita score (TC), FRS (TC) and NCEP-ATPIII FRS (TC), suggesting that these scores have only a limited power. ROC, net reclassification improvement (NRI), integrated discrimination improvement (IDI), and decision curve analysis (DCA) and Hosmer–Lemeshow goodness-of-fit test did not show clear differences between Suita score (TC) and FRS (TC). New models combining waist circumference ≥85 cm in men or proteinuria ≥1+ in women to Suita score (TC) was superior in diabetic men and women. New models could be useful to predict 3-year risk of CHD at least in Japanese population especially in diabetic population.

## Introduction

The prevalence of diabetes mellitus is increasing worldwide, particularly in Asian countries^1^. The risk for cardiovascular disease (CVD) or coronary heart diseases (CHD) in patients with diabetes is assumed to be approximately 2–3 times higher than that in patients without diabetes in Japan^2,3^ as in Western countries^4^. The Framingham Risk Score (FRS) and the SCORE risk have been reported to predict CHD in Western countries^5–8^; however, its assessment in the Japanese population has been unsuccessful^9^. Thus, original predictive models in Japan are required^10–12^.

The Suita score is a CHD-predictive model score based on the Suita study, a prospective cohort study evaluating new-onset CHD in Suita City, Osaka, Japan^9^. In 2017, the Japan Atherosclerosis Society committee revised the Japanese guideline for prevention of atherosclerotic cardiovascular diseases: risk estimation outcome has been changed from total death because of CHD, based on Nippon Data 80^2^, to new-onset CHD, based on the Suita study^9^. Hereafter, the Suita score is expected be used widely in Japan. However, several issues remain unsolved. Assessment of the Suita score in other large Japanese populations has not been performed^9^. Also, assessments in patients with diabetes have not been examined in such CHD-predictive model scores^13,14^.

In the present study, we evaluated the following: (1) assessments of FRS and Suita score in a large Japanese population using receiver operating characteristic (ROC) curve method and the Hosmer–Lemeshow test; (2) assessments of FRS and Suita scores in men and women with or without diabetes; (3) development of newly modified CHD-predictive models based on Suita score in participants with and without diabetes; (4) assessments of two scores by ROC curve, net reclassification improvement (NRI), integrated discrimination improvement (IDI), and decision curve analysis (DCA).

## Results

### Baseline characteristics and CHD onset in total participants, men, and women

The flow chart of the participants’ recruitment is shown in the Supplementary Fig. 1. The baseline characteristics of participants are shown in Table 1. The study included 35,379 participants (mean age, 62.1 years [SD 7.5]), including 14,072 men (mean age, 61.9 years [8.0]), 21,307 women (mean age, 62.3 years [7.2]), and 2,926 participants with diabetes (8.3%). The Suita score (based on total cholesterol (TC) and low-density lipoprotein cholesterol level (LDL-C)), FRS (based on TC), and NCEP-ATPIII FRS (based on TC) for total participants, men and women were shown in Table 2. Risk scores were higher in men than in women. During three years of follow up, 1,234 participants (3.49%; 589 men [4.19%], 645 women [3.03%]) developed new-onset CHD.

**Table 1:** Baseline characteristics.

Covariates	Total	Men	Women	*P*	Non-diabetic men	Diabetic men	*P*	Non-diabetic women	Diabetic women	*P*
N	35,379	14,072	21,307		12,506	1,566		19,947	1,360	
Age (years)	62.1 (7.5)	61.9 (8.0)	62.3 (7.2)	0.306	62 (8)	64 (6)	<0.001	62 (7)	64 (6)	<0.001
Height (cm)	156 (8)	164 (6)	151 (6)	<0.001	164 (6)	163 (6)	<0.001	152 (6)	151 (6)	<0.001
Body weight (kg)	57.7 (10.2)	64.7 (9.2)	53.1 (8.0)	<0.001	64.4 (9.0)	66.9 (10.2)	<0.001	52.9 (7.8)	56.8 (9.7)	<0.001
BMI (kg/m^2^)	23.5 (3.3)	24.0 (3.0)	23.2 (3.4)	<0.001	23.9 (2.9)	25.0 (3.3)	<0.001	23.0 (3.3)	25.0 (4.1)	<0.001
Waist circumference (cm)	84.0 (8.9)	85.3 (8.0)	83.1 (9.3)	<0.001	85.0 (7.9)	88.1 (8.6)	<0.001	82.8 (9.2)	87.9 (10.3)	<0.001
Systolic blood pressure (mmHg)	129 (17)	130 (17)	128 (18)	<0.001	130 (16)	134 (17)	<0.001	127 (18)	134 (17)	<0.001
Diastolic blood pressure (mmHg)	77 (10)	79 (10)	75 (10)	<0.001	79 (10)	78 (10)	0.201	75 (10)	76 (10)	<0.001
Glucose (mg/dL)	97 (17)	101 (18)	95 (15)	<0.001	97 (10)	135 (32)	<0.001	93 (9)	130 (35)	<0.001
HbA1c (%)	5.69 (0.55)	5.70 (0.61)	5.68 (0.51)	<0.05	5.56 (0.32)	6.87 (0.98)	<0.001	5.59 (0.31)	6.95 (1.03)	<0.001
TC (mg/dL)	211 (33)	204 (32)	216 (32)	<0.001	204 (32)	203 (33)	0.082	216 (32)	215 (34)	<0.05
LDL (mg/dL)	127 (29)	122 (29)	131 (29)	<0.001	123 (29)	121 (29)	<0.01	131 (29)	130 (31)	0.483
HDL (mg/dL)	62 (15)	58 (15)	65 (15)	<0.001	58 (15)	56 (15)	<0.001	65 (15)	60 (15)	<0.001
Triglyceride (mg/dL)	110 (57)	121 (63)	103 (51)	<0.001	119 (63)	131 (68)	<0.001	102 (50)	120 (58)	<0.001
AST (IU/L)	24 (8)	25 (9)	23 (7)	<0.001	25 (9)	26 (12)	0.082	23 (7)	25 (11)	<0.01
ALT (IU/L)	22 (12)	24 (13)	20 (11)	<0.001	24 (13)	28 (18)	<0.001	20 (10)	25 (17)	<0.001
γ-GTP (IU/L)	33 (37)	45 (50)	25 (22)	<0.001	44 (48)	54 (65)	<0.001	24 (21)	32 (35)	<0.001
Uric acid (mg/dL)	5.25 (1.34)	6.08 (1.29)	4.70 (1.07)	<0.001	6.11 (1.28)	5.86 (1.35)	<0.001	4.68 (1.06)	4.94 (1.18)	<0.001
Creatinine (mg/dL)	0.72 (0.18)	0.85 (0.18)	0.63 (0.13)	<0.001	0.85 (0.18)	0.84 (0.21)	<0.001	0.63 (0.12)	0.62 (0.13)	<0.001
eGFR (mL /min/1.73 m^2^)	75 (15)	74 (15)	75 (15)	<0.001	74 (14)	75 (17)	0.149	75 (15)	77 (18)	<0.01
Proteinuria (≧1+) (%)	4.3	6.0	3.3	<0.001	5.2	11.9	<0.001	2.9	7.7	<0.001
Diabetes mellitus (%)	8.3	11.1	6.4	<0.001						
Hypertension (%)	43.1	47.5	40.2	<0.001	46.0	60.0	<0.001	38.8	61.7	<0.001
Dyslipidemia (%)	53.6	50.2	55.7	<0.001	49.2	58.2	<0.001	54.7	70.8	<0.001
Anti-hypertensive drugs (%)	26.5	28.2	25.3	<0.001	26.4	42.6	<0.001	23.9	46.4	<0.001
Lipid-lowering drugs (%)	14.4	8.8	18	<0.001	7.7	17.8	<0.001	16.8	35.4	<0.001
Anti-diabetic drugs (%)	4.1	5.5	3.2	<0.001	0.0	49.7	<0.001	0.0	50.5	<0.001
Current smoker (%)	13.0	23.4	6.2	<0.001	23.1	25.9	<0.05	6.2	6.1	0.878
Alcohol drinking										
Every day (%)	18.3	38.6	4.9		38.9	36.0		5.0	3.7	
Sometimes (%)	21.5	29.8	16.0	<0.001	29.7	30.5	0.077	16.4	11.5	<0.001
Almost none (%)	60.2	70.2	84.0		31.4	33.5		78.6	84.8	

Continuous variables are expressed as mean and standard deviation. Category variables are expressed as percent. TC, LDL and HDL: total, low-density lipoprotein and high-density lipoprotein cholesterol. P values were calculated by unpaired two-tailed t-test or χ^2^ test.

**Table 2:** Values of Suita (total cholesterol and LDL-cholesterol models), Framingham Risk Score (FRS) and NCEP-ATPIII FRS scores for coronary heart disease (CHD) prediction.

Covariates	Total	Men	Women	*P*: Men vs Women	Non-diabetic men	Diabetic men	*P*: Non-diabetic vs Diabetic	Non-diabetic women	Diabetic women	*P*: Non-diabetic vs Diabetic
Number	35,379	14,072	21,307		12,506	1,566		19,947	1,360	
Suita model score (TC)	43.2 (11.7)	48.0 (11.2)	40.0 (10.9)	<0.001	47.1 (11.0)	55.3 (9.8)	<0.001	39.5 (10.8)	48.6 (9.1)	<0.001
Suita model score (LDL-C)	43.6 (10.0)	48.9 (9.1)	40.2 (9.0)	<0.001	47.9 (8.8)	56.4 (7.5)	<0.001	39.6 (8.8)	49.0 (7.1)	<0.001
FRS (TC)	6.48 (3.49)	6.51 (2.75)	6.45 (3.91)	0.086	6.20 (2.62)	9.00 (2.41)	<0.001	6.08 (3.68)	11.85 (3.09)	<0.001
NCEP-ATPIII (TC)	13.3 (3.4)	12.1 (2.5)	14.1 (3.6)	<0.001	12.0 (2.6)	12.9 (2.0)	<0.001	14.0 (3.6)	15.8 (3.0)	<0.001

Variables are expressed as mean (standard deviation). TC and LDL-C: total and low-density lipoprotein cholesterol. P values were calculated by unpaired two-tailed t-test.

### Baseline characteristics and CHD onset in men and women with and without diabetes

The study had 2,926 participants with diabetes (men [1,566], women [1,360]). Of participants without diabetes, 12,506 were men and 19,947 were women (Table 1). The following variables were higher in men and women with diabetes than those in their counterparts: age, body weight (BW), body mass index (BMI), waist circumference, systolic blood pressure (SBP), diastolic blood pressure (DBP) (in women), glucose, HbA1c, triglyceride (TG), alanine aminotransferase (ALT), γ-glutamyl transpeptidase (γ-GTP), uric acid (in women), estimated glomerular filtration rate (e-GFR) (in women), proportion of proteinuria, smoking (in men), prevalence of hypertension, hyperlipidemia, and use of anti-hypertensive, lipid-lowering, and anti-diabetic drugs. Suita score (based on TC and LDL-C), FRS (based on TC), and NCEP-ATPIII FRS (based on TC) for non-diabetic and diabetic men and non-diabetic and diabetic women were shown in Table 2. Those risk scores were all higher in diabetic men and women than in their counterparts. New-onset CHD between 2008 and 2011 was observed in men with diabetes [6.00%], men without diabetes [3.96%], women with diabetes [5.51%], and women without diabetes [2.86%], respectively.

### Assessment of FRS and Suita score in total participants, men and women

**Figure 1:**
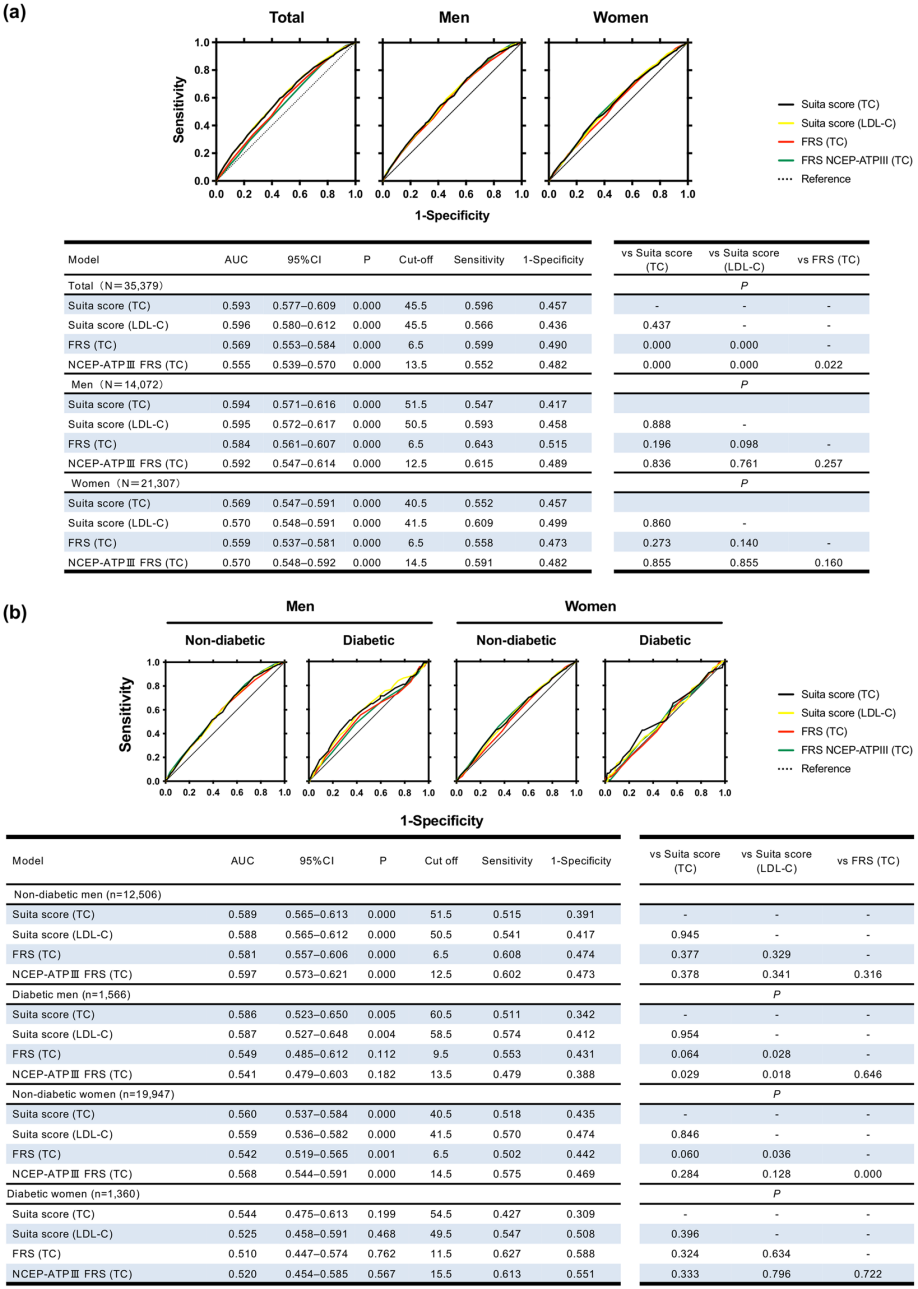
ROC curve of Suita scores (TC and LDL-C), FRS (TC), and NCEP-ATPIII FRS (TC) for coronary heart disease (CHD) prediction in total participants, men and women (**a**) and in men and women with and without diabetes. (**b**) Receiver operating characteristic (ROC) curves in Suita scores total cholesterol (TC, black line) and low-density-lipoprotein cholesterol (LDL-C, yellow line), Framingham risk score (FRS) TC (FRS TC, red line), and NCEP-ATPIII FRS TC (green line) are shown. The area under the curve (AUCs) of new-onset coronary heart disease (CHD) and cutoff values (sensitivity, 1 – specificity) and *P* values are shown in the lower left panel. *P* values for model comparisons are also shown in the lower right panel.

In ROC curves for CHD risk prediction, the areas under the curves (AUCs) of the Suita score (TC and LDL-C), FRS, and NCEP-ATPIII FRS were shown in the upper panel of Fig. 1(a) and values of AUC (95% CI) and cutoff (sensitivity, 1-specificity) were in the lower panel in Fig. 1(a). Discrimination of ROC curves was slightly different between Suita (TC) vs FRS (TC) or vs NCEP-ATPIII FRS (TC) in total participants, but that of three scores was almost equivalent when the population was divided into men and women (Fig. 1(a)).

Next, we conducted the Hosmer–Lemeshow test to evaluate the model’s calibration (Table 3). In total participants, Suita score (TC) and Suita score (LDL-C) showed a good fit, and FRS and NCEP-ATPIII FRS did not. When the population was divided into men and women, Suita, FRS and NCEP-ATPIII risk scores equivalently showed a good fit.

**Table 3:** Hosmer–Lemeshow test for coronary heart disease (CHD) prediction.

	Total			Men			Women			Non-diabetic men			Diabetic men			Non-diabetic women			Diabetic women		
	χ^2^	df	P	χ^2^	df	P	χ^2^	df	P	χ^2^	df	P	χ^2^	df	P	χ^2^	df	P	χ^2^	df	P
Suita score (TC)	4.4	8	0.824	11.9	8	0.158	11.6	8	0.171	21.8	8	0.005	8.7	8	0.368	4.9	8	0.763	8.0	8	0.432
Suita score (LDL-C)	2.9	8	0.940	9.5	8	0.300	4.0	8	0.858	11.4	8	0.180	4.9	8	0.764	5.5	8	0.701	3.7	8	0.883
FRS (TC)	22.1	8	0.005	6.4	8	0.605	2.8	8	0.946	7.0	8	0.540	7.9	8	0.440	9.4	8	0.313	2.1	8	0.977
NCEP-ATPIII FRS (TC)	18.6	8	0.017	4.4	8	0.818	4.6	8	0.798	6.8	8	0.561	3.7	8	0.880	4.2	8	0.834	6.1	8	0.637
New risk prediction model 1	3.5	8	0.903	6.6	8	0.577	13.5	8	0.096	11.3	8	0.187	7.1	8	0.524	3.4	8	0.907	7.9	8	0.448
Suita score (TC) new coefficients	8.9	8	0.350	11.7	8	0.163	8.6	8	0.377	7.6	8	0.474	13.6	8	0.092	9.0	8	0.339	14.7	8	0.066
New risk prediction model 2	10.4	8	0.238	5.6	8	0.691	8.2	8	0.411	10.7	8	0.217	5.9	8	0.653	4.2	8	0.840	9.4	8	0.309
New risk prediction model 3	9.5	8	0.303	6.8	8	0.555	1.6	8	0.991	14.4	8	0.071	5.9	8	0.655	3.7	8	0.881	15.4	8	0.051

New risk prediction model 1: Suita score (TC) + new covariates*.

New risk prediction model 2: Suita score (TC) + new coefficients + new covariates*.

New risk prediction model 3: Suita score (TC) + new coefficients + new covariates 2**.

*New covariates: Waist circumference (≥85 cm) in men and proteinuria (≥1+) in women were included.

**New covariates 2: Triglyceride, drug of diabetes, hypertension, and dyslipidemia, waist circumference (≥85 cm), and proteinuria (≥+−) in men, and triglyceride, drug of diabetes, hypertension, and dyslipidemia, waist circumference (≥90 cm), and proteinuria (≥1+) in women were included.

**Figure 2:**
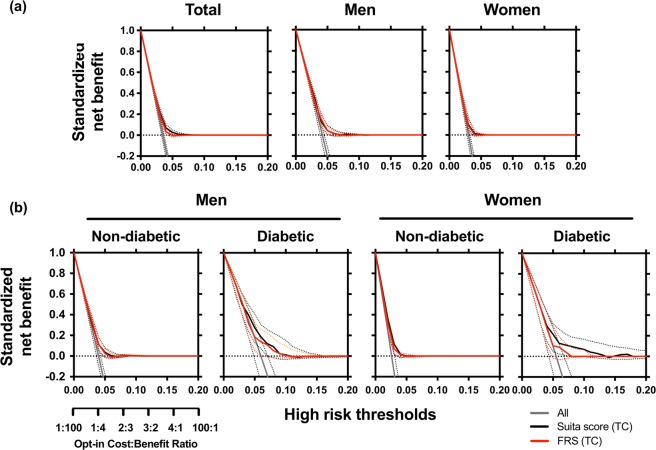
Decision curve analysis (DCA) for coronary heart disease (CHD) prediction in total participants, men and women (**a**) and in men and women with and without diabetes. (**b**) Horizontal dotted lines are net benefit of treating no participants, assuming that all would not develop CHD; gray line is net benefit of treating all participants similarly, assuming that all would develop CHD; net benefit of treating participants based on Suita score (TC) is black line and those based on FRS (TC) is red line. Dotted lines are 95% confidential intervals.

In total, men and women, NRI and IDI of ROC curves were slightly better in Suita score (TC) than in those of FRS (TC) (Tables 4 and 5). DCA of two scores was almost equally distributed (Fig. 2(a)).

**Table 4:** Net reclassification improvement (NRI) between scores for coronary heart disease (CHD) prediction.

Model	vs FRS (TC)	vs Suita Score (TC)	vs New risk prediction model 1	vs Suita score (TC) new coefficients	vs New risk prediction model 2
**Total**					
Suita score (TC)	0.205 (0.149–0.262) 0.000				
New risk prediction model 1	0.222 (0.166–0.279) 0.000	0.151 (0.095–0.208) 0.000			
Suita score (TC) new coefficients	0.296 (0.240–0.352) 0.000	0.200 (0.143–0.256) 0.000	0.189 (0.133-0.245) 0.000		
New risk prediction model 2	0.307 (0.251–0.363) 0.000	0.214 (0.158–0.271) 0.000	0.213 (0.157–0.269) 0.000	0.115 (0.059–0.171) 0.000	
New risk prediction model 3	0.347 (0.291–0.403) 0.000	0.317 (0.261–0.374) 0.000	0.271 (0.214–0.328) 0.000	0.249 (0.192–0.305) 0.000	0.236 (0.180–0.293) 0.000
**Men**					
Suita score (TC)	0.163 (0.081–0.245) 0.000				
New risk prediction model 1	0.132 (0.050–0.215) 0.002	0.152 (0.071–0.234) 0.000			
Suita score (TC) new coefficients	0.217 (0.136–0.298) 0.000	0.196 (0.114–0.277) 0.000	0.165 (0.083–0.247) 0.000		
New risk prediction model 2	0.246 (0.165-0.327) 0.000	0.209 (0.128-0.291) 0.000	0.209 (0.128-0.290) 0.000	0.151 (0.069-0.232) 0.000	
New risk prediction model 3	0.315 (0.233–0.396) 0.000	0.304 (0.222–0.386) 0.000	0.268 (0.186–0.350) 0.000	0.224 (0.142–0.307) 0.000	0.206 (0.123–0.288) 0.000
**Women**					
Suita score (TC)	0.085 (0.007–0.163) 0.034				
New risk prediction model 1	0.101 (0.023–0.179) 0.011	0.042 (−0.002–0.085) 0.059			
Suita score (TC) new coefficients	0.258 (0.180–0.335) 0.000	0.261 (0.184–0.338) 0.000	0.215 (0.137–0.292) 0.000		
New risk prediction model 2	0.248 (0.170–0.326) 0.000	0.260 (0.182–0.338) 0.000	0.267 (0.190–0.344) 0.000	0.033 (−0.036–0.101) 0.352	
New risk prediction model 3	0.335 (0.257–0.413) 0.000	0.334 (0.256–0.412) 0.000	0.327 (0.249–0.405) 0.000	0.272 (0.194–0.350) 0.000	0.269 (0.191–0.347) 0.000
**Non-diabetic men**					
Suita score (TC)	0.151 (0.062–0.241) 0.001				
New risk prediction model 1	0.103 (0.013–0.193) 0.025	0.105 (0.015–0.195) 0.022			
Suita score (TC) new coefficients	0.245 (0.158–0.333) 0.000	0.283 (0.195–0.370) 0.000	0.224 (0.135–0.312) 0.000		
New risk prediction model 2	0.253 (0.165–0.340) 0.000	0.259 (0.171–0.348) 0.000	0.285 (0.197–0.372) 0.000	0.105 (0.015–0.195) 0.022	
New risk prediction model 3	0.326 (0.237–0.415) 0.000	0.294 (0.205–0.384) 0.000	0.285 (0.196–0.374) 0.000	0.251 (0.162–0.340) 0.000	0.245 (0.156–0.334) 0.000
**Diabetic men**					
Suita score (TC)	0.259 (0.053–0.465) 0.014				
New risk prediction model 1	0.382 (0.194–0.570) 0.000	0.315 (0.142–0.488) 0.000			
Suita score (TC) new coefficients	0.281 (0.079–0.483) 0.006	0.109 (−0.099–0.317) 0.306	−0.163 (−0.366–0.040) 0.115		
New risk prediction model 2	0.342 (0.141–0.543) 0.001	0.343 (0.147–0.538) 0.001	0.139 (−0.068–0.346) 0.189	0.315 (0.142–0.488) 0.000	
New risk prediction model 3	0.469 (0.270–0.669) 0.000	0.355 (0.152–0.558) 0.000	0.208 (0.001–0.416) 0.049	0.364 (0.165–0.564) 0.000	0.299 (0.094–0.505) 0.004
**Non-diabetic women**					
Suita score (TC)	0.127 (0.044–0.210) 0.003				
New risk prediction model 1	0.139 (0.056–0.222) 0.001	0.019 (−0.022–0.059) 0.368			
Suita score (TC) new coefficients	0.293 (0.211–0.375) 0.000	0.230 (0.148–0.312) 0.000	0.196 (0.114–0.279) 0.000		
New risk prediction model 2	0.288 (0.206–0.370) 0.000	0.238 (0.156–0.320) 0.000	0.224 (0.142–0.305) 0.000	0.036 (0.001–0.071) 0.045	
New risk prediction model 3	0.342 (0.259–0.425) 0.000	0.310 (0.227–0.393) 0.000	0.306 (0.223–0.389) 0.000	0.254 (0.171–0.336) 0.000	0.238 (0.155–0.320) 0.000
**Diabetic women**					
Suita score (TC)	0.155 (−0.074–0.385) 0.184				
New risk prediction model 1	0.309 (0.113–0.505) 0.002	0.260 (0.077–0.443) 0.005			
Suita score (TC) new coefficients	0.502 (0.280–0.724) 0.000	0.461 (0.238–0.685) 0.000	0.268 (0.041–0.495) 0.021		
New risk prediction model 2	0.459 (0.230–0.688) 0.000	0.413 (0.182–0.644) 0.000	0.482 (0.260–0.704) 0.000	0.260 (0.077–0.443) 0.005	
New risk prediction model 3	0.594 (0.372–0.816) 0.000	0.586 (0.362–0.809) 0.000	0.534 (0.316–0.751) 0.000	0.300 (0.069–0.531) 0.011	0.073 (−0.160–0.306) 0.539

New risk prediction model 1: Suita score (TC) + new covariates*.

New risk prediction model 2: Suita score (TC) + new coefficients + new covariates*.

New risk prediction model 3: Suita score (TC) + new coefficients + new covariates 2**.

*New covariates: Waist circumference (≥85 cm) in men and proteinuria (≥1+) in women were included.

**New covariates 2: Triglyceride, drug of diabetes, hypertension, and dyslipidemia, waist circumference (≥85 cm), and proteinuria (≥+−) in men, and triglyceride, drug of diabetes, hypertension, and dyslipidemia, waist circumference (≥90 cm), and proteinuria (≥1+) in women were included.

**Table 5:** Integrated discrimination improvement (IDI) between scores for coronary heart disease (CHD) prediction.

Model	vs FRS (TC)	vs Suita Score (TC)	vs New risk prediction model 1	vs Suita score (TC) new coefficients	vs New risk prediction model 2
**Total**					
Suita score (TC)	0.002 (0.001–0.002) 0.000				
New risk prediction model 1	0.003 (0.002–0.003) 0.000	0.001 (0.000–0.001) 0.000			
Suita score (TC) new coefficients	0.004 (0.003–0.005) 0.000	0.002 (0.002–0.003) 0.000	0.002 (0.001–0.003) 0.000		
New risk prediction model 2	0.005 (0.004–0.006) 0.000	0.003 (0.002–0.004) 0.000	0.002 (0.002–0.003) 0.000	0.001 (0.000–0.001) 0.001	
New risk prediction model 3	0.007 (0.006–0.008) 0.000	0.005 (0.004–0.006) 0.000	0.004 (0.003–0.005) 0.000	0.002 (0.002–0.003) 0.000	0.002 (0.001–0.003) 0.000
**Men**					
Suita score (TC)	0.001 (0.000–0.002) 0.007				
New risk prediction model 1	0.002 (0.001–0.002) 0.000	0.001 (0.000–0.001) 0.002			
Suita score (TC) new coefficients	0.003 (0.002–0.004) 0.000	0.002 (0.001–0.003) 0.000	0.002 (0.001–0.003) 0.003		
New risk prediction model 2	0.004 (0.003–0.005) 0.000	0.003 (0.002–0.004) 0.000	0.002 (0.001–0.003) 0.000	0.001 (0.000–0.001) 0.004	—
New risk prediction model 3	0.006 (0.004–0.007) 0.000	0.005 (0.003–0.006) 0.000	0.004 (0.003–0.005) 0.000	0.003 (0.002–0.004) 0.000	0.002 (0.001–0.003) 0.000
**Women**					
Suita score (TC)	0.000 (0.000–0.001) 0.106				
New risk prediction model 1	0.001 (0.001–0.002) 0.000	0.001 (0.000–0.002) 0.003			
Suita score (TC) new coefficients	0.004 (0.003–0.005) 0.000	0.004 (0.003–0.005) 0.000	0.003 (0.002–0.004) 0.000		
New risk prediction model 2	0.005 (0.004–0.007) 0.000	0.005 (0.003–0.006) 0.000	0.004 (0.003–0.005) 0.000	0.001 (0.001–0.002) 0.001	—
New risk prediction model 3	0.007 (0.005–0.008) 0.000	0.007 (0.005–0.008) 0.000	0.006 (0.004–0.007) 0.000	0.003 (0.002–0.004) 0.000	0.002 (0.001–0.003) 0.001
**Non-diabetic men**					
Suita score (TC)	0.001 (0.000–0.001) 0.058				
New risk prediction model 1	0.001 (0.000–0.002) 0.016	0.000 (0.000–0.000) 0.062			
Suita score (TC) new coefficients	0.003 (0.002–0.004) 0.000	0.002 (0.001–0.003) 0.000	0.002 (0.001–0.003) 0.000		
New risk prediction model 2	0.003 (0.002–0.004) 0.000	0.003 (0.002–0.003) 0.000	0.002 (0.001–0.003) 0.000	0.000 (0.000–0.000) 0.097	—
New risk prediction model 3	0.006 (0.004–0.007) 0.000	0.005 (0.003–0.006) 0.000	0.005 (0.003–0.006) 0.000	0.003 (0.001–0.004) 0.000	0.002 (0.001–0.003) 0.000
**Diabetic men**					
Suita score (TC)	0.004 (0.001–0.006) 0.003				
New risk prediction model 1	0.009 (0.005–0.013) 0.000	0.006 (0.002–0.009) 0.001			
Suita score (TC) new coefficients	0.006 (0.002–0.009) 0.001	0.002 (−0.001–0.005) 0.250	−0.004 (0.008–0.001) 0.105		
New risk prediction model 2	0.012 (0.007–0.017) 0.000	0.008 (0.003–0.013) 0.002	0.002 (−0.001–0.006) 0.178	0.006 (0.003–0.010) 0.001	—
New risk prediction model 3	0.016 (0.010–0.023) 0.000	0.013 (0.006–0.019) 0.000	0.007 (0.001–0.012) 0.015	0.011 (0.005–0.016) 0.000	0.005 (0.000–0.009) 0.040
**Non-diabetic women**					
Suita score (TC)	0.001 (0.000–0.001) 0.001				
New risk prediction model 1	0.001 (0.000–0.001) 0.000	0.000 (0.000–0.001) 0.102			
Suita score (TC) new coefficients	0.003 (0.002–0.003) 0.000	0.002 (0.001–0.003) 0.000	0.002 (0.001–0.003) 0.000		
New risk prediction model 2	0.003 (0.002–0.004) 0.000	0.003 (0.002–0.003) 0.000	0.002 (0.002–0.003) 0.000	0.000 (0.000–0.001) 0.058	—
New risk prediction model 3	0.005 (0.004–0.006) 0.000	0.004 (0.003–0.005) 0.000	0.004 (0.003–0.005) 0.000	0.002 (0.001–0.003) 0.000	0.002 (0.001–0.003) 0.000
**Diabetic women**					
Suita score (TC)	0.001 (−0.001–0.002) 0.277				
New risk prediction model 1	0.013 (0.004–0.022) 0.005	0.012 (0.003–0.021) 0.007			
Suita score (TC) new coefficients	0.019 (0.009–0.030) 0.000	0.018 (0.008–0.029) 0.001	0.006 (−0.006–0.019) 0.323		
New risk prediction model 2	0.034 (0.015–0.052) 0.000	0.033 (0.014–0.052) 0.001	0.021 (0.006–0.035) 0.005	0.015 (0.003–0.026) 0.014	(−)
New risk prediction model 3	0.034 (0.018–0.051) 0.000	0.034 (0.017–0.050) 0.000	0.021 (0.009–0.034) 0.001	0.015 (0.004–0.026) 0.006	0.001 (−0.004–0.006) 0.815

New risk prediction model 1: Suita score (TC) + new covariates*.

New risk prediction model 2: Suita score (TC) + new coefficients + new covariates*.

New risk prediction model 3: Suita score (TC) + new coefficients + new covariates 2**.

^*^New covariates: Waist circumference (≥85 cm) in men and proteinuria (≥1+) in women were included.

^**^New covariates 2: Triglyceride, drug of diabetes, hypertension, and dyslipidemia, waist circumference (≥85 cm), and proteinuria (≥+−) in men, and triglyceride, drug of diabetes, hypertension, and dyslipidemia, waist circumference (≥90 cm), and proteinuria (≥1+) in women were included.

### Assessment of FRS and Suita score in men and women with and without diabetes

AUCs of the Suita score (TC), Suita score (LDL-C), FRS (TC) and NCEP-ATPIII FRS (TC) in non-diabetic and diabetic men and women were shown in Fig. 1(b). AUC of Suita score (TC) was slightly better in diabetic men compared to NCEP-ATPIII FRS (TC)(*P* = 0.029), however the AUCs were not different in non-diabetic men and non-diabetic and diabetic women as compared to the other scores. In Hosmer–Lemeshow test (Table 3), the values for Suita score (TC and LDL-C), FRS (TC) and NCEP-ATPIII FRS (TC) all indicated a good-fit model in men and women with or without diabetes, except for Suita score (TC) in non-diabetic men. Discrimination abilities, when assessed by NRI, IDI (Tables 4 and 5) and DCA (Fig. 2(b)), were better in Suita score (TC) than in FRS (TC), in non-diabetic and diabetic men and in non-diabetic women.

### New risk prediction model 1: Suita score (TC) + new covariates

#### Selection of new covariates

Since AUC of ROC was low in Suita score (TC) as well as in FRS (TC) and NCEP-ATPIII FRS (TC), we attempted to create a new risk prediction model by adding new covariates to Suita score (TC). Candidates of covariates in new risk prediction models are shown in Supplementary Table 1A and newly selected covariates were compared with FRS Original and the Suita score (TC) in Supplementary Table 1B. Crude odds ratio of covariates for CHD prediction was shown in Table 6. Waist circumference (≥85 cm) and BMI were significant in non-diabetic and diabetic men and proteinuria (≥± or ≥1+) was in non-diabetic and diabetic women. Calculations for Suita score (LDL-C) resulted in almost the same that in the Suita score (TC) (data not shown). Variance inflation factor (VIF) of waist circumference and proteinuria with Suita score variables were all <2.5, indicating no evidence for strong multicollinearity. Odds ratios adjusted for all covariates of Suita score, waist circumference in diabetic men (2.160; 1.290–3.617; *P* < 0.01) and proteinuria in diabetic women (3.252; 1.731–6.110; *P* < 0.01), were almost comparable with their crude odds ratios.

**Table 6:** Crude odds ratios of potential covariates for coronary heart disease (CHD) prediction.

Covariates	Non-diabetic men	*P*	Diabetic men	*P*	Non-diabetic women	*P*	Diabetic women	*P*
	Odds ratio (95%CI)		Odds ratio (95%CI)		Odds ratio (95%CI)		Odds ratio (95%CI)	
Waist circumference (≥85 or 90 cm)	1.23 (1.03–1.48)	0.022	2.18 (1.31–3.61)	0.003	1.07 (0.88–1.31)	0.496	1.17 (0.73–1.88)	0.509
BMI	1.03 (1.00–1.06)	0.041	1.09 (1.03–1.15)	0.004	1.01 (0.98–1.04)	0.470	1.06 (1.00–1.11)	0.046
AST	1.00 (0.99–1.01)	0.541	1.01 (0.99–1.02)	0.518	1.01 (1.00–1.02)	0.254	1.00 (0.98–1.02)	0.963
ALT	1.00 (0.99–1.01)	0.805	1.00 (0.99–1.01)	0.672	1.00 (0.99–1.01)	0.577	1.00 (0.98–1.01)	0.700
γ-GTP	1.00 (1.00–1.00)	0.187	1.00 (1.00–1.00)	0.682	1.00 (1.00–1.01)	0.247	1.00 (0.99–1.01)	0.592
Uric acid	1.07 (1.00–1.15)	0.057	1.07 (0.92–1.25)	0.361	1.01 (0.93–1.09)	0.815	1.10 (0.90–1.33)	0.359
Proteinuria (≥±)	1.11 (0.87–1.42)	0.394	1.62 (1.04–2.53)	0.034	1.52 (1.19–1.94)	0.001	1.95 (1.12–3.39)	0.018
Proteinuria (≥1+)	1.32 (0.92–1.90))	0.132	1.32 (0.73–2.39)	0.353	1.64 (1.11–2.44)	0.014	3.32 (1.81–6.08)	0.000
FPG	1.01 (1.00–1.02)	0.021	1.00 (0.99–1.00)	0.561	1.00 (0.99–1.01)	0.879	1.00 (0.99–1.01)	0.785
HbA1c	1.53 (1.16–2.03)	0.003	0.99 (0.80–1.23)	0.933	1.25 (0.95–1.64)	0.113	1.21 (1.00–1.46)	0.045
Alcohol drinking (everyday, sometimes, almost none)	0.97 (0.87–1.08)	0.620	0.87 (0.68–1.12)	0.273	1.12 (0.95–1.32)	0.176	1.01 (0.62–1.65)	0.966

#### Developing new risk prediction model 1

We implemented a new risk prediction model as follows. The β coefficients calculated using multiple logistic regression in diabetic men were Suita score 0.3, waist circumference 7, and intercept −5. Then, the formula for modified Suita score in diabetic men was 0.3 × Suita score (TC) + 7 × waist circumference. Although the intercept was omitted in this formula for simplification, the intercept was used to calculate the probability of CHD onset. In the same way, the β coefficients calculated using multiple logistic regression in women with diabetes were Suita model score 0.1, proteinuria 12, and intercept −4. Thus, the formula for modified Suita score in diabetic women was 0.1 × Suita score (TC) + 12 × proteinuria ≥1+ (0 or 1). In non-diabetic men, the formula = 0.3 × Suita score (TC) + 1.2 × waist circumference ≥85 cm (0 or 1), whereas that for non-diabetic women was 0.2 × Suita score (TC) + 4.3 × proteinuria ≥1+ (0 or 1). The results for Suita score (LDL-C) were almost the same as those for Suita score (TC) (data not shown).

#### Hosmer–Lemeshow goodness-of-fit test

Hosmer–Lemeshow test (Table 3) for Suita score (TC) new covariates indicated a good-fit model in total, men and women, and in non-diabetic and diabetic men and women.

#### ROC curve, NRI, IDI and DCA

**Figure 3:**
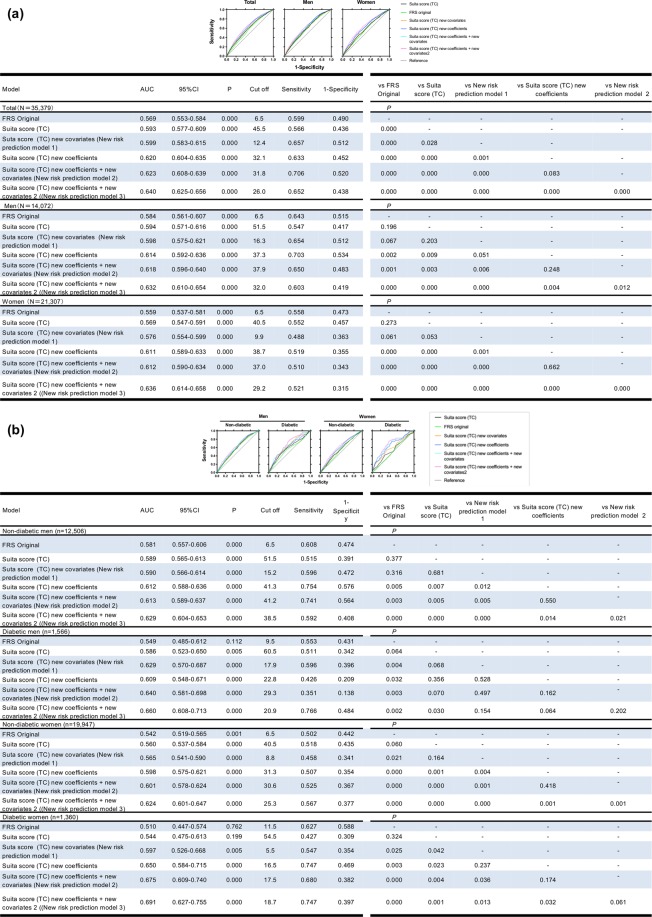
ROC curves of FRS, Suita score (TC) and its modified scores for coronary heart disease (CHD) prediction in total participants, men and women (**a**) and in men and women with and without diabetes. (**b**) Receiver operating characteristic (ROC) curves in FRS (light green lines), Suita score (TC) (black lines), New risk prediction model 1 (orange lines), Suita score (TC) new coefficients (blue lines), New risk prediction model 2 (light blue lines), and New risk prediction model 3 are shown. The area under the curve (AUCs) of new-onset coronary heart disease (CHD) and cutoff values (sensitivity, 1 – specificity) and P values are shown in the lower left panel. P values for model comparisons are also shown in the lower right panel. New risk prediction model 1: Suita score (TC) + new covariates*, New risk prediction model 2: Suita score (TC) new coefficients + new covariates*, New risk prediction model 3: Suita score (TC) new coefficients + new covariates 2**, *new covariates: Waist circumference (≥85 cm) in men and proteinuria (≥1+) in women were included, **new covariates 2: Triglyceride, drug of diabetes, hypertension, and dyslipidemia, waist circumference (≥85 cm), and proteinuria (≥+−) in men, and triglyceride, drug of diabetes, hypertension, and dyslipidemia, waist circumference (≥90 cm), and proteinuria (≥1+) in women were included.

**Figure 4:**
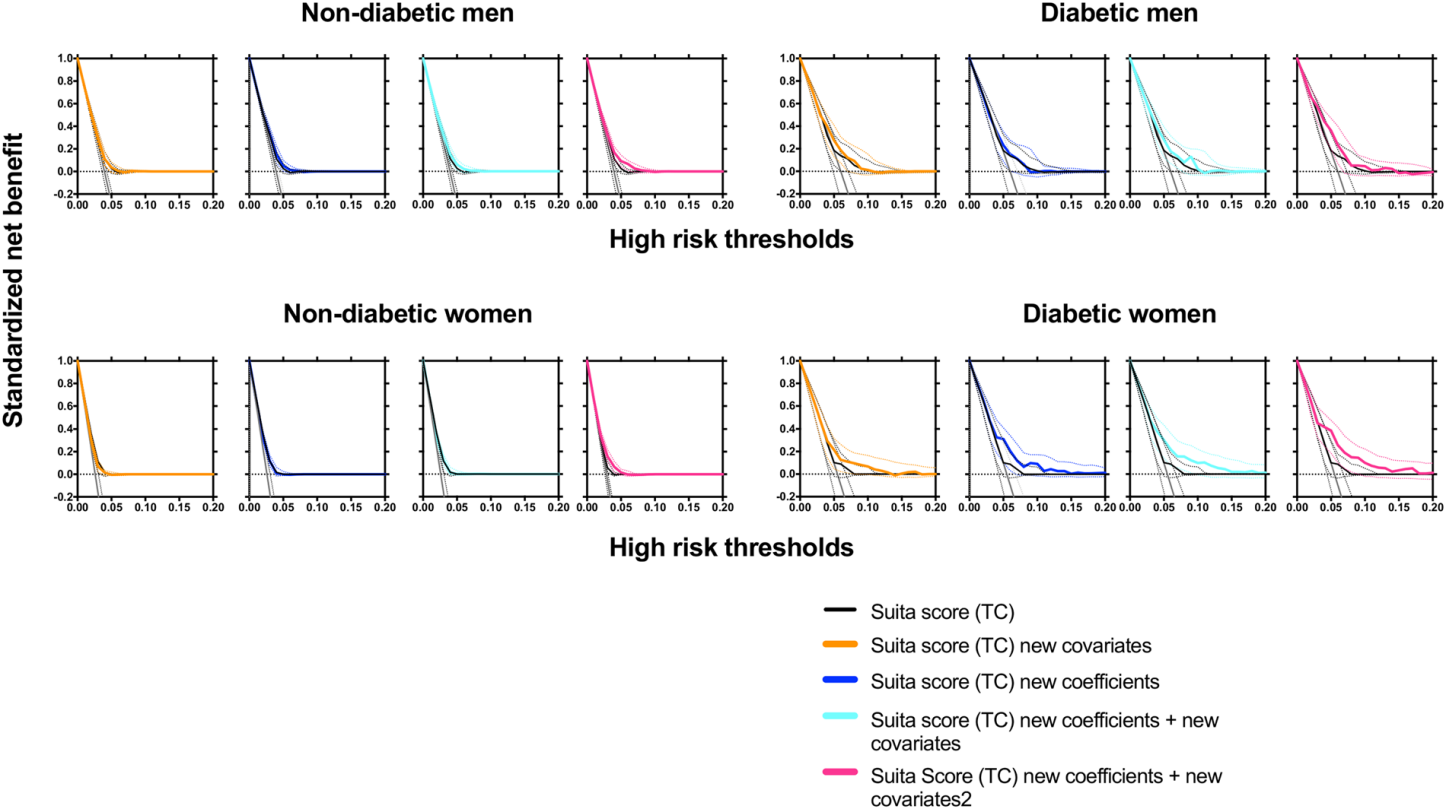
Decision curve analysis (DCA) for coronary heart disease (CHD) prediction in men and women with and without diabetes. Horizontal dotted lines are net benefit of treating no participants, assuming that all would not develop CHD; gray line is net benefit of treating all participants similarly, assuming that all would develop CHD; net benefit of treating participants based on Suita score (TC) is black line and those based on New risk prediction model 1 (orange), Suita score (TC) new coefficients (blue lines), New risk prediction model 2 (light blue lines), and New risk prediction model 3 (light pink lines)are also shown. Dotted lines are 95% confidential intervals. (New risk prediction model 1: Suita score (TC) + new covariates*, New risk prediction model 2: Suita score (TC) new coefficients + new covariates*, New risk prediction model 3: Suita score (TC) new coefficients + new covariates 2**, *new covariates: Waist circumference (≥85 cm) in men and proteinuria (≥1+) in women were included, **new covariates 2: Triglyceride, drug of diabetes, hypertension, and dyslipidemia, waist circumference (≥85 cm), and proteinuria (≥+−) in men, and triglyceride, drug of diabetes, hypertension, and dyslipidemia, waist circumference (≥90 cm), and proteinuria (≥1+) in women were included.

We weighed the discrimination ability of the new risk prediction model by comparing AUC (Delong test), NRI, IDI and DCA. Addition of new covariates, waist circumference or proteinuria, to Suita score (TC) significantly improved AUCs in total and women (Fig. 3(a)), however showed a borderline difference (P = 0.068) in diabetic men and a significant difference in diabetic women (P = 0.042) (Fig. 3(b)). Meanwhile, the overall category-free NRI and IDI of Suita score (TC) new covariates indicated significance or borderline differences as compared to Suita score (TC) in total, men and women, and in non-diabetic and diabetic men and diabetic women (Tables 4 and 5). The DCA (Fig. 4) showed that curves of Suita score (TC) new covariates had a good discrimination in diabetic men and women, but not in non-diabetic men and women.

#### Probability of new-onset CHD

The probability of new-onset CHD for 3 years using Suita score (TC or LCL-C) covariates were calculated in diabetic men and women. If waist circumference was positive in men, the probability of CHD was increased approximately 2-fold, whereas if proteinuria was positive in women, the probability was increased approximately 3-fold (Supplementary Tables 2 and 3).

### New risk prediction model 2: Suita score (TC) new coefficients + new covariates

#### New coefficients for Suita score (TC) covariates

We developed another new risk model in which all the variables included in the Suita score were reassessed to obtain better model performance.β-coefficients of Suita score (TC) variables^9^ such as age, TC, HDL-C, SBP, current smoking, HbA1c and e-GFR were recalculated in the-sex specific models to obtain best fit models.

In non-diabetic men: Suita score (TC) new coefficients = 0.44 × age (years) + 0.05 × SBP (mmHg) + 0.01 × TC (mg/dL) − 0.05 × HDL-C (mg/dL) − 0.96 × current smoking (0 or 1) − 0.07 × eGFR + 2.5 × HbA1C (%).

In diabetic men: Suita score (TC) new coefficients = 0.38 × age (years) + 0.04 × SBP (mmHg) + 0.001 × TC (mg/dL) − 0.03 × HDL-C (mg/dL) + 1.38 × current smoking (0 or 1) − 0.12 × eGFR + 0.10 × HbA1C (%).

In non-diabetic women: Suita score new coefficients = 0.36 × age (years) + 0.08 × SBP (mmHg) − 0.06 × TC (mg/dL) + 0.02 × HDL-C (mg/dL) − 2.73 × current smoking (0 or 1) + 0.03 × eGFR + 1.2 × HbA1C (%).

In diabetic women: Suita score new coefficients = 0.48 × age (years) − 0.01 × SBP (mmHg) − 0.11 × TC (mg/dL) + 0.00 × HDL-C (mg/dL) + 1.35 × current smoking (0 or 1) − 0.11 × eGFR + 2.64 × HbA1C (%).

#### Suita score (TC) new coefficients + new covariates

New risk prediction model 2 was implemented adding waist circumference in men and proteinuria ≥1 in women to Suita score (TC) new coefficients scores as follows.

In non-diabetic men: Suita score (TC) new coefficients + new covariates = 0.98 × Suita score (TC) new coefficients + 1.11 × waist circumference ≥85 cm (0 or 1).

In diabetic men: Suita score (TC) new coefficients + new covariates = 0.96 × Suita score new coefficients + 7.40 × waist circumference ≥85 cm (0 or 1).

In non-diabetic women: Suita score (TC) new coefficients + new covariates = 0.98 × Suita score new coefficients + 4.48 × proteinuria ≥1+ (0 or 1).

In diabetic women: Suita score (TC) new coefficients + new covariates = 0.96 × Suita score new coefficients + 11.1 × proteinuria ≥1+ (0 or 1).

#### Hosmer–Lemeshow goodness-of-fit test

As shown in Table 3, performance of Suita Score (TC) new coefficients and Suita Score (TC) new coefficients + covariates were good in total, men and women, were also good when divided to non-diabetic and diabetic men and women.

#### ROC curve, NRI, IDI, and DCA

As compared Suita score (TC), Suita score (TC) new coefficients with or without new covariates significantly improved AUCs in total, men and women (Fig. 3(a)), in non-diabetic men and non-diabetic and diabetic women (Fig. 3(b)). The overall category-free NRI and IDI of Suita score (TC) new coefficients with or without new covariates also showed improvement in total, men and women, non-diabetic men and non-diabetic and diabetic women. (Tables 4 and 5). DCA indicated that curve discrimination of Suita score (TC) new coefficients with or without covariates was improved compared to those of Suita score (TC) in diabetic men and women (Fig. 4).

### New risk prediction model 3: Suita score (TC) new coefficients + new covariates 2

#### Suita score (TC) new coefficients + new covariates 2

Finally, to obtain the best risk prediction models, we reconsidered a set of new comprehensive candidates of covariates: sex, age, BW, BMI, waist circumference, waist circumference (≥85 cm in men and ≥90 cm in women), SBP, DBP, FPG, HbA1c, AST, ALT, γ-GTP, HDL-C, LDL-C, triglyceride, uric acid, serum creatinine, eGFR, proteinuria, proteinuria (≥±), proteinuria (≥1+), smoking habits, drinking habits, drug information of diabetes, hypertension, and dyslipidemia (Supplementry Table 1A). We selected the newly selected covariates as new covariates 2 (Supplementry Table 1B).

In detail, new models are as follows; Age, SBP, TC, HDL, HbA1c, current smoking, and e-GFR (Suita score (TC) new coefficients), adding waist circumference ≥85 cm, the use of drug of diabetes, hypertension, and dyslipidemia, triglyceride, and proteinuria ≥+− (new covariates 2) in men: meanwhile, Age, SBP, TC, HDL, HbA1c, current smoking, and e-GFR(Suita score (TC) new coefficients), adding proteinuria ≥1+, the use of drug of diabetes, hypertension, and dyslipidemia, triglyceride, and waist circumference ≥90 cm (new covariates 2) in women, respectively. Next, we calculated β-coefficients of Suita score (TC) new coefficients and new covariates 2 in the-sex specific models to obtain best fit models.

In non-diabetic men: Suita score (TC) new coefficients + new covariates 2 = 0.86 × Suita score (TC) new coefficients + 0.52 × waist circumference ≥85 cm (0 or 1) + 3.74 × the use of drug of hypertension (0 or 1) + 3.22 × the use of drug of dyslipidemia (0 or 1) + 0 × triglyceride + 0.16 × proteinuria ≥+− (0 or 1).

In diabetic men: Suita score (TC) new coefficients + new covariates 2 = 0.87 × Suita score new coefficients +8.02 × waist circumference ≥85 cm (0 or 1)+ 1.08 × the use of drug of hypertension(0 or 1), +1.98 × the use of drug of diabetes (0 or 1), −1.88 × the use of drug of dyslipidemia, (0 or 1) −0.03 × triglyceride + 3.84 × proteinuria ≥+− (0 or 1).

In non-diabetic women: Suita score (TC) new coefficients + new covariates 2 = 0.75 × Suita score new coefficients + 3.42 × proteinuria ≥1 (0 or 1) + 5.03 × the use of drug of hypertension(0 or 1), +1.44 × the use of drug of dyslipidemia(0 or 1), + 0 × triglyceride −1.01 × waist circumference ≥90 cm (0 or 1).

In diabetic women: Suita score (TC) new coefficients + new covariates 2 = 0.9 × Suita score new coefficients + 11.03 × proteinuria ≥1 (0 or 1) + 1.54 × the use of drug of hypertension(0 or 1), +2.38 × the use of drug of diabetes (0 or 1) + 1.30 × the use of drug of dyslipidemia,(0 or 1), −0.02 × triglyceride +0.29 × waist circumference ≥90 cm (0 or 1).

#### Hosmer–Lemeshow goodness-of-fit test

As shown in Table 3, performance of Suita score (TC) new coefficients + covariates 2 were good in total, men, and women, in non-diabetic and diabetic men and women.

#### ROC curve, NRI, IDI, and DCA

As compared FRS or Suita score (TC), Suita score (TC) new coefficients + new covariates 2 significantly improve AUCs in total, men and women (Fig. 3(a)), also significantly improved AUCs in non-diabetic and diabetic men and women (Fig. 3(b)). As compared FRS or Suita score (TC), the overall category-free NRI and IDI of Suita score (TC) new coefficients with new covariates 2 also showed improvement in total, men, women and in non-diabetic and diabetic men and women (Tables 4 and 5). DCA indicated that curve discrimination of Suita score (TC) new coefficients with covariates 2 was improved compared to those of Suita score (TC) in diabetic men and women (Fig. 4).

## Discussion

In the current study, we assessed how accurately the risk scores which were originally developed for predicting 10-year risk of CHD^9^ could predict 3-year risk of CHD in a large Japanese population. We obtained three major findings in the study. First, ROC curve distribution of Suita score (TC) was slightly different between vs FRS (TC) and NCEP-ATPIII FRS (TC) in total participants, but was almost equivalent when the population was divided into men and women. The AUCs were consistently low in Suita score (TC), FRS (TC) and NCEP-ATPIII FRS (TC), suggesting that these risk scores have only a limited power for CHD risk prediction in Japanese population. Second, ROC curve distribution, NRI and IDI, and DCA did not show clear differences between Suita score (TC) and FRS (TC)/ NCEP-ATPIII FRS (TC) in men and women with or without diabetes. Third, new models combining waist circumference ≥85 cm or proteinuria ≥1+ to Suita score (TC) or Suita score new coefficients (TC) (new risk prediction model 2) was proven better than its respective models, when assessed by AUC, NRI, IDI, and Hosmer–Lemeshow test. In addition, the AUCs of new risk prediction model 3 (Suita score (TC) new coefficients + new covariates 2) were also superior to those of the FRS and Suita scores in total, men and women, but the superiority was observed especially when population were subdivided to non-diabetic and diabetic men and women. Two new models could be useful to predict 3-year risk of CHD at least in Japanese population especially in diabetic population.

Epidemiological studies in Japan, such as Nippon Data 80^2,3^ and the Hisayama Study^9^, reported that current risk prediction models are not adequate. Nakai *et al*.^15^ reported that, the expected CHD mortality rate was rather higher than the observed CHD onset in Nippon Data 80. The guidelines for prevention of atherosclerotic cardiovascular diseases in 2017, which had been established based on the Suita study, are increasingly used in Japan. However, the issue whether Suita score can be used satisfactory in real practice remains not fully evaluated. To the best of our knowledge, our study is the first to assess the utility of Suita score using a large Japanese population and clarified the limited ability discussed below. Nishimura *et al*.^9^ reported: FRS might overestimate CHD incidence in a Japanese general population, while Suita score (TC or LDL-C) could improve the estimation power. In our Japanese population, discrimination of Suita score (TC) for estimating CHD was slightly better compared to FRS (TC) and NCEP-ATPIII FRS (TC) in total participants, but the performance was comparable, when the population was divided into men and women. Also, Hosmer–Lemeshow test indicated that FRS (TC) did not fit in total participants but did in men and women subgroups as well as in FRS (TC), suggesting that both scores may be equivalent in the sex-specific risk prediction (Table 3). We assessed discrimination ability of Suita score (TC) in non-diabetic and diabetic men and women. ROC curve distribution, NRI and IDI, and DCA were partially better in Suita score (TC) than FRS (TC) or NCEP-ATPIII FRS (TC) in several subcategory, but the results were not consistent among ROC, NRI and IDI, and DCA. Because the CHD predictive models of Japanese especially in general population are limited^10,12^, we further sleeked a risk prediction model in participants with or without diabetes as discussed below.

Since the power of Suita score (TC) for CHD risk prediction was limited, we tried to create new models. Interestingly, combining waist circumference ≥85 cm or proteinuria ≥1+ to Suita score (TC) or Suita score new coefficients (TC) was proven superior to its respective models (new risk prediction model 2). Gender difference in covariates for CHD risk prediction has been discussed^16^.

Waist circumference is a useful and convenient substitute index for visceral fat, which is associated with insulin resistance^17^. In general, visceral fat accumulates more easily in men, and subcutaneous fat accumulates more easily in women^18,19^. Therefore, adding waist circumference tended to increase AUC in diabetic men (Suita score (TC) new covariates vs Suita score (TC), *P* = 0.068, Fig. 3(b)) is consistent with that of previous studies^18,19^. We reported that men with increased waist circumference was closely related to endothelial dysfunction, an early feature of CHD^20,21^. Insulin resistance is the other important residual risk factor of CHD^22^. We previously proved that endothelial dysfunction was correlated with individual metabolic risk components, such as diabetes, dyslipidemia, hypertension or visceral obesity, but most strongly with clustering of the components under a condition with low insulin sensitivity^23^. Although markers for visceral fat obesity and/or insulin resistance are not included in Suita score^19^, waist circumference is reported to be one such marker. Therefore, adding waist circumference to the CHD predictive model can be valuable.

In contrast, proteinuria was recently reported to be an independent cardiovascular disease (CVD) risk factor, which is different from e-GFR^24^. Irie *et al*.^25^ reported that the combination of proteinuria and e-GFR was an efficient predictor of CVD in the Japanese general population. Accordingly, adding proteinuria and including e-GFR to the Suita score were not contradicted in their report. Meanwhile, in our study, adding proteinuria to the Suita score demonstrated different results between men and women with diabetes, although the reasons for this remain unclear. Irie *et al*.^25^ also found that the age-adjusted relative CHD risk because of proteinuria was 2.90 (95% CI, 1.94–4.34) and 4.54 (95% CI, 2.93–7.04) in men and women, suggesting that the relative CVD risk with proteinuria was higher in women. The sex difference of CVD risk with proteinuria remains undetermined and further study is needed to clarify the issue. The utility of urinary microalbumin levels rather than considering proteinuria in the model might be more informative for CHD risk assessment^26,27^. We could not measure microalbuminuria due to limitation of cost. Alternatively, we compared the impact of proteinuria (±) and proteinuria (1+) on ROC: in diabetic men, Suita score (TC) AUC = 0.586 (95%CI 0.523–0.650, *P* = 0.005), plus proteinuria (±), AUC = 0.598 (95%CI 0.539–0.657, *P* < 0.001), plus proteinuria (+) AUC = 0.586 (0.524–0.648, *P* = 0.005); in diabetic women, Suita score (TC) AUC = 0.544 (95%CI, 0.475–0.613, *P* = 0.199), plus proteinuria (±) AUC = 0.573 (0.504–0.643, *P* = 0.033, plus proteinuria (+) AUC = 0.597 (0.526–0.668, *P* = 0.005). AUC was increased by addition of proteinuria (±) greatly in men, but slightly in women. Utility of dipstick proteinuria and albuminuria for CHD risk prediction need to be discussed in further studies^28^.

We also created new risk prediction model 3 (Suita score (TC) new coefficients + new covariates 2), in which the AUCs of were also superior to those of the FRS and Suita scores in total, men and women, but the superiority was observed especially when population were subdivided to non-diabetic and diabetic men and women. Addtiion of triglyceride, and drug of diabetes, hypertension, and dyslipidemia may increase the power of CHD prediction in thesubdivided groups. There are risk prediction scores for diabetic patients in the world^29–31^ and in Japan^11^. As compared to c-statics in CHD (male 0.65, female 0.71) in UK database^30^, CHD (0.74) in Hong Kong Diabetes Registry (HKDR)^31^, CHD (0.725) in JJ risk enjine^11^, AUC (c-statics) of current new Suita score (TC) (0.660 in diabetic men, 0.691 in diabetic women) was still low. It is suggested that including other potential covariates such as nutritional^16^ and genetic risk factors^32,33^ or family history of premature myocardial infarction^34^ to the model will be beneficial for a better risk prediction.

### Our study has several limitations

First, the duration of observation was 3 years, which was shorter than that in previous studies. Also, the number of censored cases was large, and the reason of censoring could not be obtained by limitations of study design. It could give bias of samplings, which considerably influence statistical analysis. Although time-to-event analysis could not be obtained in the current analysis, we are planning to elongate periods of observations (~7 years) in the same population to validate our risk prediction model. Second, past medical history of diseases was determined only by medical interview, and evaluations were not fully objective. To compensate for the medical interview, the study physicians conducted a physical evaluation of each subject and reassessed their medical history. Third, the definition of CHD differed between the Suita study and ours. In the Suita study, CHD was defined as acute myocardial infarction, CHD followed by coronary artery bypass or angioplasty, and sudden cardiac death within 24 h after acute onset of symptoms, not including angina^9^. Meanwhile, we defined CHD as angina or myocardial infarction. The difference in definition of CHD can cause a difference in the prevalence of new-onset CHD. In fact, the prevalence of CHD was higher in our study. The new onset CHD cases could serve as an index of performance of Suita score, if this scoring is compared with percentage stenosis of coronary arteries. Our recent study indicated that FRS used in the current study was not largely associated with significant coronary stenosis (r = 0.197, *P* = 0.006) and Gensini score, an index of coronary atherosclerosis severity (r = 0.200, *P* = 0.005)^35^ on computed tomography angiography. Associations between CHD onset and coronary atherosclerosis need to be evaluated in terms of risk prediction scores in future studies. Fourth, types of medicines and diabetes duration could not be evaluated fully in an annual health check program. In diabetes, the past state of glycemic control, so-called “metabolic memory” or “legacy effect” can strongly affect onset of CHD, but that information cannot be obtained by our study, limiting our interpretation. Fifth, the assessment of our new prediction models should be done in other Japanese diabetic population databases. The features of the Suita study and annual health check program (SHCG) (this study) are compared in Supplementary Table 4, indicating two different Japanese population.

### Conclusion

We demonstrated the differential assessment of the Suita score in a large population of Japanese men and women with and without diabetes. Adding waist circumference and proteinuria to the Suita score in men and women with diabetes, respectively, resulted in better discrimination. Because the costs are limited in an annual health check program, development of a low-cost and convenient marker is very important. We have shown a benefit of new Suita scores for CHD prediction as compared to original Suita score in diabetic participants. However, the discrimination ability of the scores remains unsatisfactory and thus future studies are warranted to develop better models.

## Methods

### Study population

This study was conducted as a part of the ongoing “Research on the Positioning of Chronic Kidney Disease in Specific Health Check and Guidance in Japan (SHCG)” project^36,37^. To determine external validations of the Suita and FRS scores, we analyzed data from participants who were followed up longitudinally from 2008 to 2011 based on the SHCG. The annual health check program, SHCG, was started by the Japanese government in 2008, targeting early diagnosis and intervention for metabolic syndrome in Japanese citizens aged 40–74 years. If necessary, public health nurses provide lifestyle interventions in participants who are at high risk for metabolic syndrome^38^. Data were collected and confirmed by the NPO Japan Clinical Research Support Unit (Tokyo, Japan).

### Primary outcome and study design

The primary outcome was defined as new-onset CHD (angina or myocardial infarction). The flow chart of the participants’ recruitment is shown in the Supplementary Fig. 1. Of 667,218 participants with available data at baseline in 2008, 109,653 were available for longitudinal data until 2011. Of these, 41,301 were selected after 68,352 were excluded because of the missing data. We excluded 2673 participants because of baseline history of CHD (n = 1767) and/or cerebrovascular disease (n = 1065) and 2935 because of missing data on CHD in 2009, 2010, and 2011. Participants with TG ≥400 mg/dL (n = 314) were also excluded^39^. Finally, 35,379 participants (14,072 men; 21,307 women) were selected and analyzed.

### FRS (original or National Cholesterol Education Program Adult Treatment Panel III [NCEP-ATPIII] version) and Suita score (TC or LDL-C version)

We calculated the FRS^5^ and NCEP-ATPIII FRS^6^, using data on age, sex, TC, high-density lipoprotein cholesterol level (HDL-C), SBP, smoking, DBP (FRS), diabetes (FRS)^5^, and anti-hypertensive drug intake (NCEP-ATPIII)^6^. Suita scores were calculated similarly to that in the Suita study (TC or LDL-C version), using data on age, sex, TC or LDL-C, HDL-C, SBP, DBP, smoking, diabetes, and e-GFR^9^.

### Covariates

Trained staff measured height, BW, waist circumference, and SBP and DBP using a standard sphygmomanometer or an automated device on the right arm after resting for 5 min in a sitting position based on the recommendations of the Japanese Ministry of Health, Labor and Welfare^40^. The participants described age; sex; smoking and alcohol intake; anti-hypertensive, anti-diabetic, and anti-dyslipidemia medication intake; and history of CHD, cerebrovascular disease, and kidney dysfunction on a questionnaire. CHD was defined as angina or myocardial infarction; cerebrovascular disease, as cerebral hemorrhage or cerebral infarction; and kidney dysfunction, as chronic renal failure or need for dialysis. Blood samples were collected after overnight fasting. Centrifuged samples were analyzed by automatic clinical chemistry analyzer within 24 h of sampling. All blood samples were analyzed at local, rather than central, laboratories. Analyses were conducted by the methods for laboratory tests recommended by the Japan Society of Clinical Chemistry^37^. The analyzed factors were the following: fasting plasma glucose (FPG), HbA1c (JDS or National Glycohemoglobin Standardization Program [NGSP]), AST, ALT, γ-GTP, HDL-C, LDL-C, TG, uric acid (UA) and serum creatinine. In addition, HbA1c (JDS) was converted to HbA1c (NGSP), which was calculated as JDS + 0.4%^41^. e-GFR was calculated as follows: e-GFR (men) = 194 × Scr^−1.094^ × age^−0.287^ and e-GFR (women) = e-GFR (men) × 0.739^42^. Urine was tested using the dipstick method on spot urine specimens gathered from participants in the early morning after overnight fasting. Proteinuria was defined as positive ≥1+ and negative− or± ^40^. Alcohol consumption subgroups were classified as those who drink every day, drink sometimes, and drink almost never. Diabetes mellitus was determined by American Diabetes Association criteria^43^: FPG ≥ 126 mg/dL, HbA1c-NGSP ≥ 6.5%, or use of anti-diabetic medications. Hypertension was defined as SBP ≥ 140 mmHg, or DBP ≥ 90 mmHg, or anti-hypertensive medication intake. Dyslipidemia was defined as LDL-C 140 ≥ mg/dL, HDL-C ≤ 40 mg/dL, TG ≥ 150 mg/dL, or anti-dyslipidemic drug intake. If necessary, TC was calculated by the Friedewald’s method^39^, and participants whose TG was ≥400 mg/dL were excluded.

### Statistical analyses

#### Subgroups of participants

We divided the participants into four subgroups of men and women with and without diabetes and assessed the risk prediction power of the Suita and FRS scores.

#### ROC curve, NRI, IDI and DCA

The risk score of new-onset CHD was calculated as the sum of the individual new coefficients in the Suita score (TC and LDL-C) and original and NCEP-ATPIII FRS. We then assessed the predictive performance for the risk score by drawing an ROC curve, by calculating the AUCs for ROC curve, and sensitivity, specificity, and positive and negative predictive values for cutoff values^44^. The cutoff value was determined by the maximum AUC value, and AUCS were compared by the DeLong test^45^.

#### Hosmer–Lemeshow test

We compared the predicted and observed incidence of new-onset CHD in each decile of risk score and performed the Hosmer–Lemeshow test to assess goodness-of-fit of the model. If *P* < 0.05, the model was regarded as no goodness of fit^46^.

#### New risk prediction model 1: Suita score (TC) +new covariates

Selection of covariates: Finally, to create a better predictive model, particularly in participants with diabetes, we evaluated the impact of adding covariates to the Suita score. As residuals in the Suita score, we selected potential covariates measured in the SHCG: abdominal obesity (waist circumference ≥85 cm in men, >90 cm in women)^47^, BMI,AST, ALT, γ-GTP, UA, proteinuria (≥+−),proteinuria (≥1+), FPG, HbA1c, and alcohol consumption (drinking every day, sometimes, and never). Crude odds ratio of new-onset CHD was calculated individually by adding a selected covariate to the Suita score, and the covariate (s) with the highest crude odds ratio was selected.

Assessment of covariates added to the Suita score: We assessed the influence of newly added covariates by comparing the adjusted odds ratio in multiple logistic regression. For an additional covariate, multicollinearity was checked with the VIF. If multicollinearity was less extent (VIF ≤ 2.5)^48^, we implemented the new covariate in the new model as bellow.

Developing modified Suita score: Using multiple logistic regression, we calculated the β-coefficients by adding new covariates to the Suita score. We multiplied the value 10 times and rounded the respective β-coefficients to create new coefficients primarily used β-coefficients:

$$lo{g}_{e}(\frac{p}{1-p})={\beta }_{0}+{\beta }_{1}{x}_{1}+{\beta }_{2}{x}_{2}\,+\,\cdots \,+\,{\beta }_{k}{x}_{k}$$

Finally, we calculated the modified Suita score by adding a new covariate to the original Suita score.

Reclassification of new model of diabetes: Pencina *et al*.^49^ reported that NRI and IDI are valuable to evaluate the modified model’s ability of discrimination, which was assessed by evaluating AUC changes^45^. Finally, we drew a DCA of the Suita score and modified the score. DCA is the method of calculating the net benefit, which means the true positive value is subtracted from the false positive value^50^.

Calculation of the CHD probability by multiple logistic regression models: We calculated the probability of CHD for 3 years by multiple logistic regression based on the modified Suita score:

$$\hat{p}=\frac{1}{1+exp(-\sum _{i=0}^{p}{\beta }_{i}{X}_{i})}$$

(β = coefficient)^51,52^.

#### New risk prediction model 2: Suita score (TC) new coefficients +new covariates

Recalculation of regression coefficients: We developed the new prediction model by adding appropriate covariates to the Suita score as above [Suita score (TC) new covariates]. However, if the model performance of the original Suita score might be insufficient, which would possibly just come from “inaccurate” regression coefficients in the Suita score. Therefore, we develop another risk model in which all the coefficients included in the Suita score were entered as explanatory variables and check its model performance. The β-coefficients for CHD prediction, calculated using multiple logistic regression, were obtained for variables used in the Suita score (TC) [age, SBP, TC, HDL-C, HbA1c, eGFR, and current smoking]. Suita score (TC) new coefficients models were made based on results of discrimination (ROC, NRI, IDI and DCA) and calibration (Hosmer–Lemeshow test).

Adding covariates on Suita score (TC) new coefficients score: As in Suita score (TC) new covariates, other covariates other than Suita score (TC) new coefficients were tested. Crude odds ratio for new-onset CHD was calculated individually by adding a selected covariate to the Suita score (TC) new coefficients model, and the covariate(s) with the highest crude odds ratio was selected to the model. Furthermore, additionally develop risk models in which above coefficients included in the Suita score plus other covariates were entered as explanatory variables and check their model performance.

#### New risk prediction model 3: Suita score (TC) new coefficients +new covariates 2

Adding covariates 2 on Suita score (TC) new coefficients score: Finally, to obtain the best risk prediction models, we reconsidered a set of new comprehensive candidates of covariates: sex, age, BW, BMI, waist circumference, waist circumference (≥85 cm in men and ≥90 cm in women), SBP, DBP, FPG, HbA1c, AST, ALT, γ-GTP, HDL-C, LDL-C, triglyceride, uric acid, serum creatinine, eGFR, proteinuria, proteinuria (≥±), proteinuria (≥1+), smoking habits, drinking habits, drug information of diabetes, hypertension, and dyslipidemia (Supplementry Table 1A). We selected the newly selected covariates as new covariates 2 (Supplementry Table 1B) and the model performance were also assessed by Hosmer–Lemeshow test and ROC curve, NRI, IDI, and DCA.

#### Statistical applications

The data were expressed as mean ± standard deviation or %. For a two-group comparison, unpaired two-tailed *t*-test or Mann–Whitney *U* test were used for parametric or nonparametric distribution variables, respectively. The X^2^ test was used for non-continuous variables and the DeLong test was for AUC. *P* < 0.05 was considered statistically significant. Assessment in subgroups was completed by the model’s ability for discrimination (ROC curve and AUC)^44^ and calibration [the Hosmer–Lemeshow test]). For statistical analyses, we used SPSS (version 24.0; SPSS, Inc., Chicago, IL, USA) for basic descriptive statistics and R statistical package (R version 3.3.2) for ROC (pROC), NRI and IDI (PredictABEL), DCA (rmda) and Hosmer–Lemeshow test (ResourceSelection).

### Ethics approval and consent to participate

All procedures performed in studies involving human participants were in accordance with the ethical standards of the institutional and/or national research committee at which the studies were conducted (Fukushima Medical University; IRB Approval Number #1485, #2771) and with the 1964 Helsinki declaration and its later amendments or comparable ethical standards. This study was conducted according also to the Ethical Guidelines for Medical and Health Research Involving Human Subjects enacted by MHLW of Japan [http://www.mhlw.go.jp/file/06-Seisakujouhou-10600000-Daijinkanboukouseikagakuka/0000069410.pdf and http://www.mhlw.go.jp/file/06-Seisakujouhou-10600000-Daijinkanboukouseikagakuka/0000080278.pdf]. In the context of the guideline, the investigators shall not necessarily be required to obtain informed consent, but we made public information concerning this study on the web [http://www.fmu.ac.jp/univ/sangaku/data/koukai_2/2771.pdf] and ensured the opportunities for the research subjects to refuse utilizing their personal information.

## Supplementary information

Supple Table 1-4, Supple Fig 1

Supple Table 2

Supple Table 3

## Data Availability

The datasets during and/or analyzed during the current study available from the corresponding author on reasonable request.
