# Supplementary material for: New risk prediction model of coronary heart disease in participants with and without diabetes: Assessments of the Framingham risk and Suita scores in 3-year longitudinal database in a Japanese population
Source: Sci Rep. 2019 Feb 26;9:2813. doi: 10.1038/s41598-019-39049-w (PMC6391401; doi:10.1038/s41598-019-39049-w)

New risk prediction model of coronary heart disease in participants with and without diabetes: Assessments of the Framingham risk and Suita scores in 3-year longitudinal database in a Japanese population

Hiroyuki Hirai, Koichi Asahi, Satoshi Yamaguchi, Hirotaka Mori, Hiroaki Satoh, Kunitoshi Iseki, Toshiki Moriyama, Kunihiro Yamagata, Kazuhiko Tsuruya, Shouichi Fujimoto, Ichiei Narita, Tsuneo Konta, Masahide Kondo, Yugo Shibagaki, Masato Kasahara, Tsuyoshi Watanabe and Michio Shimabukuro

Supplementary Table 1. Candidates and selection of covariates in new risk prediction models

A. Candidates of covariates in new risk prediction models

Sex, age, BW, BMI, waist circumference, Waist circumference (≥ 85 cm) in men, Waist circumference (≥ 90 cm) in women, SBP, DBP, FPG, HbA1c, AST, ALT, γ-GTP, HDL-C, LDL-C, triglyceride, uric acid, serum creatinine, eGFR, proteinuria, proteinuria (≥±) , proteinuria (≥1+), smoking habits, drinking habits, drug information of diabetes, hypertension, and dyslipidemia.

B. Covariates in FRS Original, the Suita score (TC) and new risk prediction models

**Model**	**Total**
FRS Original	Age, Sex, SBP and DBP, TC, HDL-C, Smoking, and Diabetes
Suita score (TC)	Age, Sex, SBP and DBP, TC, HDL-C, Smoking, Diabetes, and e-GFR
Suita score (TC) + new covariates (New risk prediction model 1)	Suita score (TC) + Waist circumference (≥85 cm), and Proteinuria (≥1+)
Suita score (TC) new coefficients	Age, Sex, SBP, TC, HDL-C, Smoking, HbA1c, and e-GFR
Suita score (TC) new coefficients + new covariates (New risk prediction model 2)	Suita score (TC) new coefficients + Waist circumference (≥85 cm), and Proteinuria (≥1+)
Suita score (TC) new coefficients + new covariates 2 (New risk prediction model 3)	Suita score (TC) new coefficients + Waist circumference (≥85 cm), Proteinuria (≥+-), Triglyceride, and Drug of diabetes, hypertension, and dyslipidemia
**Model**	**Men**
FRS Original	Age, Sex, SBP and DBP, TC, HDL-C, Smoking, and Diabetes
Suita score (TC)	Age, Sex, SBP and DBP, TC, HDL-C, Smoking, Diabetes, and e-GFR
Suita score (TC) + new covariates (New risk prediction model 1)	Suita score (TC) + Waist circumference (≥85 cm)
Suita score (TC) new coefficients	Age, Sex, SBP, TC, HDL-C, Smoking, HbA1c, and e-GFR
Suita score (TC) new coefficients + new covariates (New risk prediction model 2)	Suita score (TC) new coefficients + Waist circumference (≥85 cm)
Suita score (TC) new coefficients + new covariates 2 (New risk prediction model 3)	Suita score (TC) new coefficients + Waist circumference (≥85 cm), Triglyceride, Drug of diabetes, hypertension, and dyslipidemia, and Proteinuria (≥+-)
**Model**	**Women**
FRS Original	Age, Sex, SBP and DBP, TC, HDL-C, Smoking, and Diabetes
Suita score (TC)	Age, Sex, SBP and DBP, TC, HDL-C, Smoking, Diabetes, and e-GFR
Suita score (TC) + new covariates (New risk prediction model 1)	Suita score (TC) + Proteinuria (≥1+)
Suita score (TC) new coefficients	Age, Sex, SBP, TC, HDL-C, Smoking, HbA1c, and e-GFR
Suita score (TC) new coefficients + new covariates (New risk prediction model 2)	Suita score (TC) new coefficients + Proteinuria (≥1+)
Suita score (TC) new coefficients + new covariates 2 (New risk prediction model 3)	Suita score (TC) new coefficients + Proteinuria (≥1+), Triglyceride, Drug of diabetes, hypertension, and dyslipidemia, and Waist circumference (≥90 cm)

See methods in detail

Supplementary Table 2. Probability of new onset CHD estimated by Suita score (TC) in diabetic men and women

Waist over 85 cm	Modified Suita model score (TC)	probability (%)
yes	33.7	16.6
yes	31.6	14.0
yes	30.4	12.7
yes	30.1	12.4
yes	29.5	11.8
yes	28.9	11.2
yes	28.6	10.9
yes	28.3	10.6
yes	28.0	10.4
yes	27.7	10.1
yes	27.4	9.8
yes	26.8	9.4
yes	26.5	9.1
yes	26.2	8.9
yes	25.9	8.7
yes	25.6	8.4
yes	25.3	8.2
yes	25.0	8.0
yes	24.7	7.8
yes	24.4	7.6
yes	24.1	7.4
yes	23.8	7.2
yes	23.5	7.0
yes	23.2	6.8
yes	22.9	6.7
yes	22.6	6.5
yes	22.3	6.3
yes	22.0	6.1
yes	21.7	6.0
yes	21.4	5.8
yes	21.1	5.7
yes	20.8	5.5
yes	20.5	5.4
yes	20.2	5.2
yes	19.9	5.1
yes	19.6	5.0
yes	19.3	4.8
yes	19.0	4.7
yes	18.7	4.6
yes	18.4	4.4
yes	18.1	4.3
yes	17.5	4.1
yes	17.2	4.0
yes	16.9	3.9
yes	16.6	3.8
yes	16.3	3.7
yes	16.0	3.6
yes	15.4	3.4
yes	14.8	3.2
yes	13.6	2.9
yes	13.3	2.8

Waist over 85 cm	Modified Suita model score (TC)	probability (%)
no	25.8	8.0
no	23.1	6.3
no	22.8	6.2
no	22.5	6.0
no	21.9	5.7
no	21.6	5.5
no	21.3	5.4
no	21.0	5.3
no	20.7	5.1
no	20.4	5.0
no	20.1	4.8
no	19.8	4.7
no	19.5	4.6
no	19.2	4.5
no	18.9	4.3
no	18.6	4.2
no	18.3	4.1
no	18.0	4.0
no	17.7	3.9
no	17.4	3.8
no	17.1	3.7
no	16.8	3.6
no	16.5	3.5
no	16.2	3.4
no	15.6	3.2
no	15.3	3.1
no	14.7	3.0
no	14.4	2.9
no	14.1	2.8
no	13.5	2.7
no	13.2	2.6
no	12.9	2.5
no	12.3	2.4
no	12.0	2.3
no	11.4	2.2
no	10.8	2.1
no	10.5	2.0
no	9.9	1.9
no	9.6	1.8
no	8.4	1.7
no	7.2	1.5
no	6.3	1.4
no	5.1	1.2

Proteinuria over(1+)	Modified Suita model score (TC)	probability (%)
yes	19.1	18.0
yes	18.8	17.4
yes	18.7	17.3
yes	18.6	17.1
yes	18.5	16.9
yes	18.4	16.7
yes	18.3	16.5
yes	18.0	16.0
yes	17.8	15.6
yes	17.7	15.4
yes	17.5	15.1
yes	17.4	14.9
yes	17.3	14.7
yes	17.2	14.6
yes	17.1	14.4
yes	17.0	14.2
yes	16.8	13.9
yes	16.6	13.6
yes	16.5	13.4
yes	16.4	13.3
yes	16.3	13.1
yes	16.1	12.8
yes	16.0	12.7
yes	15.9	12.5
yes	15.8	12.4
yes	15.3	11.7
yes	14.6	10.7
yes	14.2	10.2

Proteinuria over(1+)	Modified Suita model score (TC)	probability (%)
no	7.1	6.3
no	6.9	6.2
no	6.8	6.1
no	6.7	6.0
no	6.6	5.9
no	6.4	5.8
no	6.3	5.7
no	6.1	5.6
no	6.0	5.5
no	5.8	5.4
no	5.7	5.3
no	5.5	5.2
no	5.4	5.1
no	5.2	5.0
no	5.1	4.9
no	5.0	4.8
no	4.8	4.7
no	4.6	4.6
no	4.5	4.5
no	4.3	4.4
no	4.0	4.3
no	3.8	4.2
no	3.6	4.1
no	3.5	4.0
no	3.4	3.9
no	3.1	3.8
no	2.9	3.7
no	2.6	3.6
no	2.5	3.5
no	2.2	3.4
no	2.0	3.3
no	1.7	3.2
no	1.1	2.9

The probabilities were calculated based on multiple logistic regressions.

Supplementary Table 3. Probability of new onset CHD estimated by Suita score (LDL-C) in diabetic men and women.

Waist over 85 cm	Modified Suita model score (TC)	probability (%)
yes	30.7	14.2
yes	30.4	13.8
yes	30.1	13.4
yes	28.9	11.9
yes	28.6	11.5
yes	28.3	11.2
yes	28.0	10.8
yes	27.7	10.5
yes	27.4	10.2
yes	27.1	9.9
yes	26.8	9.6
yes	26.5	9.3
yes	26.2	9.0
yes	25.9	8.7
yes	25.6	8.4
yes	25.3	8.2
yes	25.0	7.9
yes	24.7	7.7
yes	24.4	7.4
yes	24.1	7.2
yes	23.8	7.0
yes	23.5	6.8
yes	23.2	6.6
yes	22.9	6.3
yes	22.6	6.1
yes	22.3	5.9
yes	22.0	5.8
yes	21.7	5.6
yes	21.4	5.4
yes	21.1	5.2
yes	20.8	5.1
yes	20.5	4.9
yes	20.2	4.7
yes	19.9	4.6
yes	19.6	4.4
yes	19.3	4.3
yes	19.0	4.2
yes	18.7	4.0
yes	18.4	3.9
yes	18.1	3.8
yes	17.8	3.6
yes	17.2	3.4
yes	16.3	3.1
yes	15.7	2.9
yes	14.2	2.4

Waist over 85 cm	Modified Suita model score (TC)	probability (%)
no	22.5	6.5
no	21.9	6.1
no	21.6	5.9
no	21.3	5.7
no	21.0	5.5
no	20.7	5.4
no	20.4	5.2
no	20.1	5.0
no	19.8	4.9
no	19.5	4.7
no	19.2	4.6
no	18.9	4.4
no	18.6	4.3
no	18.3	4.1
no	18.0	4.0
no	17.7	3.9
no	17.4	3.7
no	17.1	3.6
no	16.8	3.5
no	16.5	3.4
no	16.2	3.3
no	15.9	3.2
no	15.6	3.1
no	15.3	3.0
no	15.0	2.9
no	14.7	2.8
no	14.4	2.7
no	14.1	2.6
no	13.8	2.5
no	13.5	2.4
no	12.9	2.3
no	12.6	2.2
no	12.3	2.1
no	11.7	2.0
no	11.1	1.9
no	10.8	1.8
no	10.5	1.7
no	9.6	1.6
no	9.3	1.5

Proteinuria over(1+)	Modified Suita model score (TC)	probability (%)
yes	18.4	16.4
yes	18.3	16.2
yes	18.2	16.1
yes	18.1	15.9
yes	17.9	15.6
yes	17.8	15.4
yes	17.7	15.3
yes	17.6	15.1
yes	17.5	15.0
yes	17.4	14.8
yes	17.3	14.7
yes	17.2	14.5
yes	17.1	14.4
yes	17.0	14.2
yes	16.9	14.1
yes	16.8	13.9
yes	16.7	13.8
yes	16.6	13.7
yes	16.5	13.5
yes	16.4	13.4
yes	16.3	13.2
yes	16.2	13.1
yes	16.1	13.0
yes	15.8	12.6
yes	15.7	12.4
yes	15.6	12.3
yes	14.4	10.8

Proteinuria over(1+)	Modified Suita model score (TC)	probability (%)
no	7.0	6.1
no	6.6	5.8
no	6.4	5.7
no	6.3	5.6
no	6.1	5.5
no	6.0	5.4
no	5.9	5.3
no	5.6	5.2
no	5.4	5.1
no	5.3	5.0
no	5.1	4.9
no	4.9	4.8
no	4.8	4.7
no	4.6	4.6
no	4.4	4.5
no	4.2	4.4
no	3.9	4.3
no	3.7	4.2
no	3.6	4.1
no	3.4	4.0
no	3.1	3.9
no	2.9	3.8
no	2.7	3.7
no	2.4	3.6
no	2.1	3.5

The probabilities were calculated based on multiple logistic regressions.

Supplementary Table 4. Comparisons between the Suita study and the current study

Study	Suita study	Comprehensive annual health check
Aim	Evaluation of the new onset CHD	Early detection and prevention of
Study design	Longitudinal urban observational	observational cohort study
The method of selection	Randomized	Wills of participants
Study population	Suita city, Osaka, Japan	All over, Japan
Numbers	5521 (men 2796, women 2725)	35,379 (men 14,072, women 21,307)
Age, years (Mean ± SD)	men 56.1 ± 13.3, women 54.5 ± 12.9	men 61.93 ± 8.01 , women 62.25 ±
Dulation of periods, Years	11.8	3
New onset of the CHD, Numbers	179 (all)	1,234 (men 589, women 645)
Definition of the new onset CHD	Acute myocardial infarction ,CHD followed by coronary artery bypass , CHD followed by angioplasty, sudden cardiac death within 24 hours after the onset of acute symptoms	Acute myocardial infarction , Angina
The incidence of the CHD (numbers/1000 person-years)	2.81 (all)	3.49% (all)<br>4.19% (men)<br>3.03% (women)<br>3.96% (non-diabetic men)<br>6.00% (diiabetic men)<br>2.86% (non-diabetic women)<br>5.51% (diabetic women)
The prevalence of diabetes at baseline	men 6.0%, women 5.8%	men 11.1%, women 6.4%

Supplement Fig. 1. Flow chart of the participants' recruitment

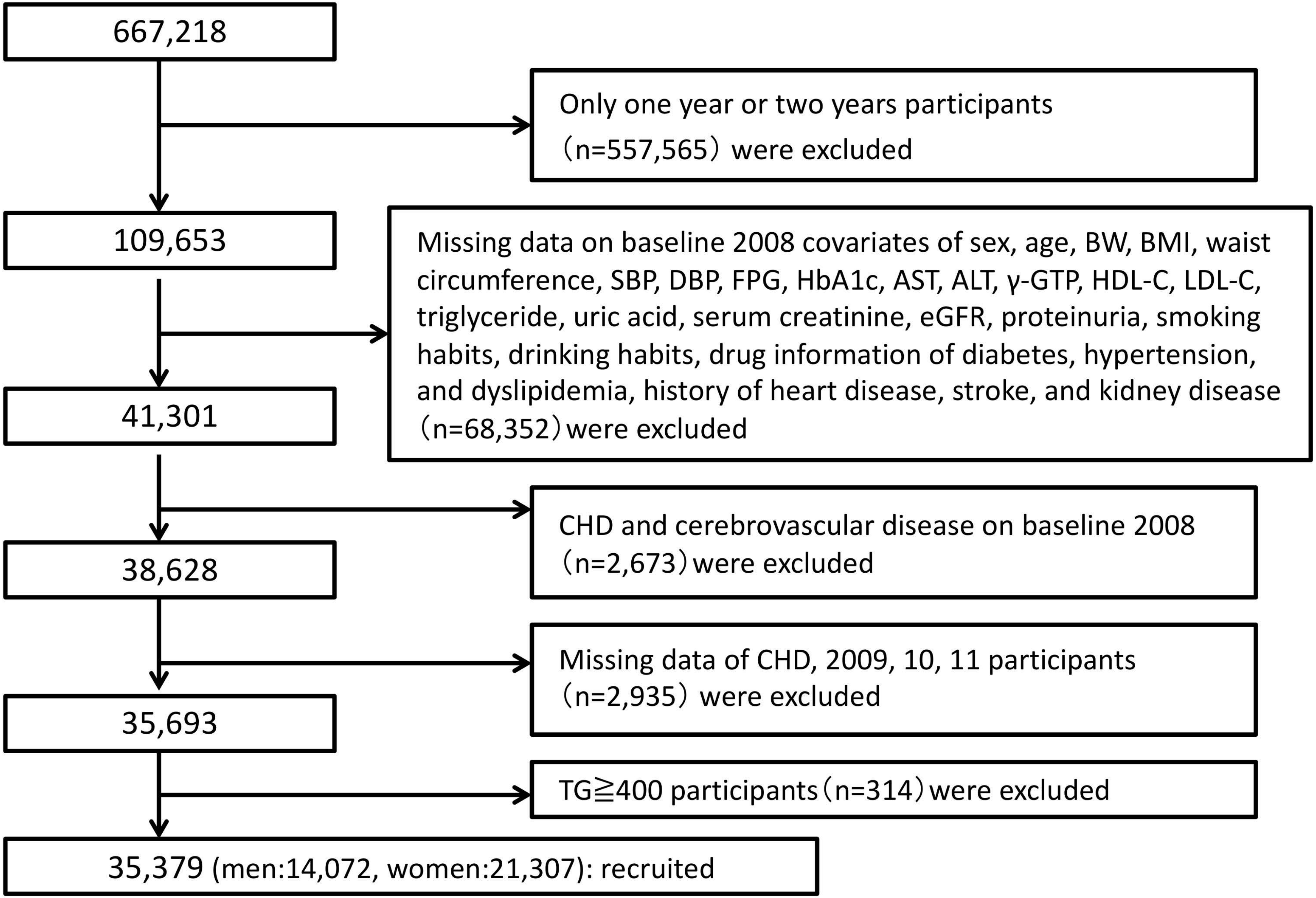

Supplement: Supplementary file 1 — Supple Table 1-4, Supple Fig 1 [file 41598_2019_39049_MOESM1_ESM.pdf]
